# *Pseudomonas aeruginosa* elastase cleaves a C-terminal peptide from human thrombin that inhibits host inflammatory responses

**DOI:** 10.1038/ncomms11567

**Published:** 2016-05-16

**Authors:** Mariena J. A. van der Plas, Ravi K. V. Bhongir, Sven Kjellström, Helena Siller, Gopinath Kasetty, Matthias Mörgelin, Artur Schmidtchen

**Affiliations:** 1 Division of Dermatology and Venereology, Department of Clinical Sciences Lund, Lund University, BMC, Tornavägen 10, Lund SE-22184, Sweden; 2 Department of Biochemistry and Structural Biology, Center for Molecular Protein Science, Lund University, PO Box 124, Lund SE-22362, Sweden; 3 Division of Infection Medicine, Department of Clinical Sciences Lund, Lund University, BMC, Tornavägen 10, Lund SE-22184, Sweden; 4 Dermatology and Venereology, Skane University Hospital, Lasarettsgatan 15, Lund SE-22185, Sweden; 5 Dermatology, LKCMedicine, Nanyang Technological University, 59 Nanyang Drive, Singapore 636921, Singapore

## Abstract

*Pseudomonas aeruginosa* is an opportunistic pathogen known for its immune evasive abilities amongst others by degradation of a large variety of host proteins. Here we show that digestion of thrombin by *P. aeruginosa* elastase leads to the release of the C-terminal thrombin-derived peptide FYT21, which inhibits pro-inflammatory responses to several pathogen-associated molecular patterns *in vitro* and *in vivo* by preventing toll-like receptor dimerization and subsequent activation of down-stream signalling pathways. Thus, *P. aeruginosa* ‘hijacks' an endogenous anti-inflammatory peptide-based mechanism, thereby enabling modulation and circumvention of host responses.

## Abstract (web summary)

Neutrophil elastase cleaves thrombin generating anti-inflammatory peptides. Here the authors show that cleavage of thrombin by *Pseudomonas aeruginosa* elastase generates a peptide that prevents TLR dimerization and signaling, interfering with the inflammatory response to avoid host defense.

## Graphical abstract

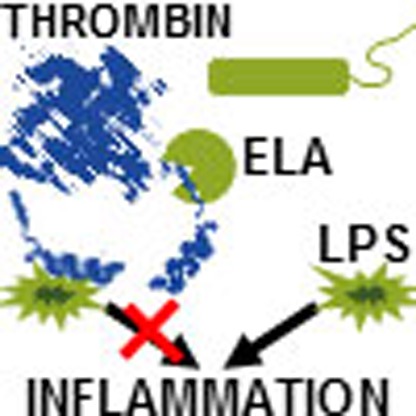

P*seudomonas aeruginosa* is a Gram-negative opportunistic pathogen associated with severe infections often seen in immuno-compromised and critically ill patients as well as individuals with cystic fibrosis, burns or chronic wounds. These disabling infections can cause a severely reduced quality of life for patients and are associated with delayed healing^1^,^2^ and increased morbidity and mortality^3^,^4^,^5^. *P. aeruginosa* is commonly known for its immune evasive abilities by the production of various extracellular and also cell-associated virulence factors, such as proteases, exotoxins, alginate, lipopolysaccharide (LPS) and rhamnolipids, that can amongst others alter coagulation and vascular permeability^6^,^7^, activate zymogens such as the highly abundant plasma protein prothrombin^7^, and degrade a large variety of proteins such as extracellular matrix components^8^,^9^, cytokines^10^, complement factors and coagulation factors^11^. Reports describing these findings for *P. aeruginosa* refer to degradation of proteins often as a loss of function with diminished immune responses as a consequence. However, in a broader biological perspective, protein digestion leads to the direct formation of many fragments that may exert novel functions. In agreement, proteolytic activity by endogenous enzymes may result in the generation of host defence peptides (HDPs) and in some cases activated proteins, which may not only exert antimicrobial effects, but also affect cytokine release, complement activation and coagulation during inflammation^12^,^13^. Degradation of human thrombin by neutrophil elastase, for example, generates amongst other antimicrobial fragments, an 18 amino acid long C-terminal peptide (HVFRLKKWIQKVIDQFGE, denoted HVF18)^14^, which is capable of dampening inflammation^15^. This observed multifunctionality of HDPs is in line with the fact that many classical antimicrobial peptides, such as cathelicidins and defensins, have been shown to mediate numerous biological effects, including chemotaxis, phagocytosis and other inflammatory responses^16^,^17^,^18^. However, recent evidence shows that classical antimicrobial peptides, such as LL-37, likely exert little or no antimicrobial activity *in vivo*, while the immunomodulatory effects dominate at physiological concentrations^19^,^20^.

As *P. aeruginosa* proteases could resemble the activity of neutrophil elastase, we hypothesized that *P. aeruginosa* can mimic endogenous anti-inflammatory mechanisms by creating ‘HDP-like' immunomodulatory peptides, that can aid this bacterial species in circumvention of host responses. To test this hypothesis and based on the above findings and considerations, we investigated whether *P. aeruginosa* proteases can form C-terminal-derived bioactive peptides from thrombin.

Here we show that *P. aeruginosa* elastase cleaves a C-terminal-derived peptide from thrombin, designated FYT21, which inhibits activation of the transcription factors nuclear factor (NF)κB and activator protein 1 (AP-1), and the subsequent release of pro-inflammatory cytokines in response to LPS *in vitro* and *in vivo*. *In vivo* imaging demonstrated that the production of reactive oxygen species in response to LPS is reduced by FYT21 as well. Fluorescence-activated cell sorting (FACS), slot blot assays and electron microscopy studies showed that FYT21 binds both to LPS and cell membranes thereby preventing receptor dimerization and activation. The actions of FYT21 are not specific to LPS, as the peptide also inhibits NFκB/AP-1 activation in response to lipoteichoic acid (LTA), zymosan A, oligonucleotides (ODNs) and the cytokine tumor necrosis factor (TNF)-α. In agreement, FYT21 inhibits cytokine release and NFκB/AP-1 activation in response to bacterial conditioned medium. Moreover, *P. aeruginosa* elastase reduces inflammatory cytokine production *in vivo*, showing a proof of principle that the bacterial enzyme indeed reduces pro-inflammatory responses under physiological conditions. Finally, FYT21 is present in chronic wound fluids and on leukocytes derived therefrom.

Taken together, the observation that *P. aeruginosa* can degrade an abundant host protein thereby leading to the release of a peptide that dampens inflammation illustrates a novel concept of pathogen–host interactions, where mimicking an endogenous anti-inflammatory mechanism may aid these bacteria in evading host responses.

## Results

### Isolation of thrombin-derived C-terminal peptides

**Figure 1:**
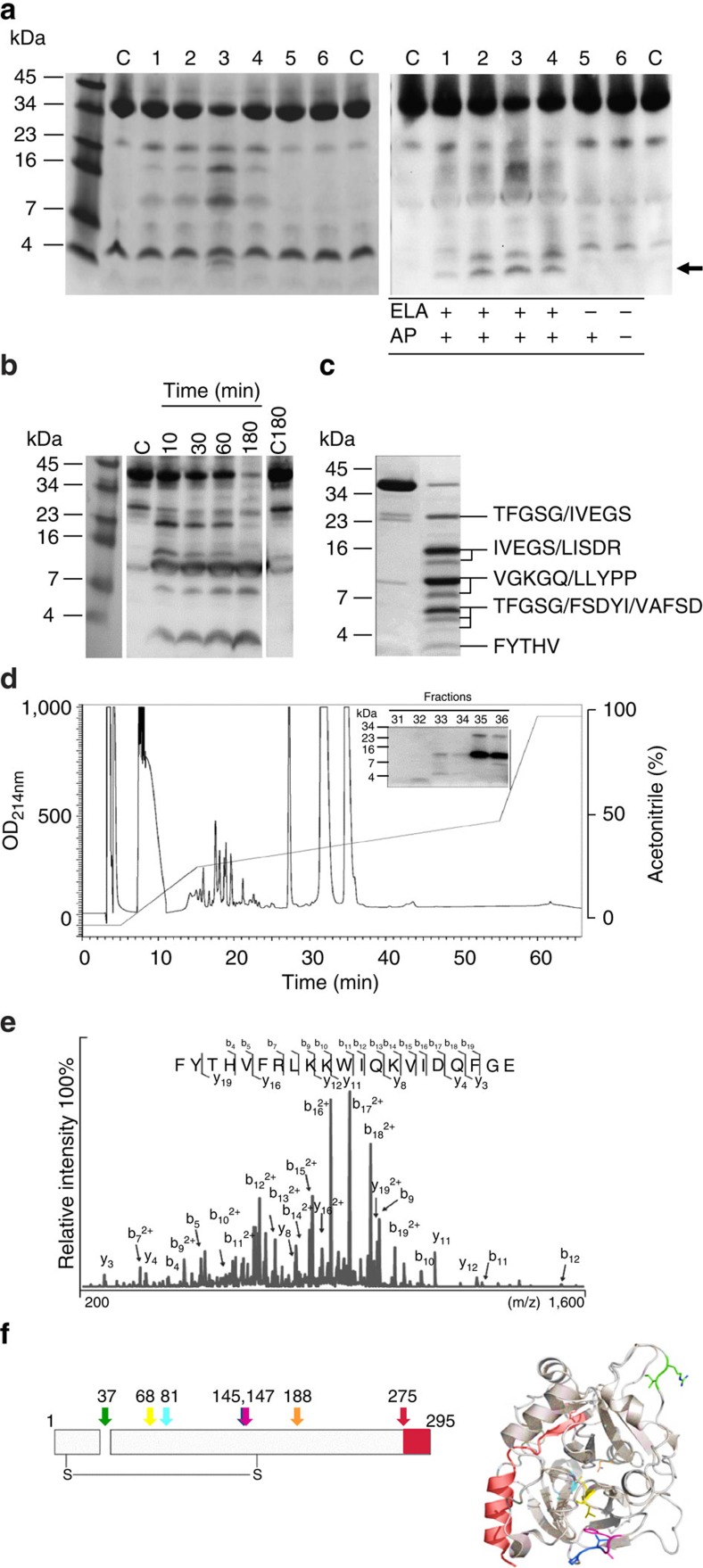
Isolation of the C-terminal-derived thrombin peptide FYT21. (**a**) Human α-thrombin was digested with CM from several protease producing (1:PAO1, 2–4: clinical isolates) and non-producing (5: PAOB1; 6: clinical isolate) strains for 1 h at 37 °C and analysed by SDS–PAGE (left) and western blotting (right). Thrombin alone was used as a control (c). The arrow indicates peptides of similar molecular mass as the endogenous HVF18, which are only formed in the presence of CM of elastase producing strains. (**b**) Thrombin was digested with purified *P. aeruginosa* elastase for various periods of time and analysed using western blotting. Thrombin alone was used as a control directly (C) or after 3 h incubation at 37 °C (C180). (**c**) Thrombin fragments obtained after 1 h digestion, followed by separation by SDS–PAGE, were subjected to N-terminal sequencing. (**d**) HPLC profile of digested thrombin. The insert shows the fractions containing C-terminal epitopes of thrombin. The full sequence of the peptide in fraction 32 was obtained using Orbitrap analysis (**e**). (**f**) A 2D (showing the inter-chain disulphide bond) and a 3D model of thrombin depicting all cleavage sites of elastase. Each colour represents one cleavage site. The C-terminal peptide FYT21 is depicted in red. Of note, the sequences TFGSG and IVEGS represent the N termini of the light and heavy chains, respectively.

To investigate whether *P. aeruginosa* proteases cleave human thrombin, we incubated thrombin with conditioned medium (CM) from various protease producing and non-producing strains for 1 h at 37 °C, ran the digest on a Tris-Tricine gel and used it for western blotting. The results showed that CM of strains PAO1, 3.1, 15159 and 10.5 (numbers 1–4), producing the metalloprotease elastase, cleaved thrombin, thereby releasing a C-terminal peptide of <4 kDa (Fig. 1a, arrow), which is comparable in size to the thrombin-derived peptide HVF18, formed by human neutrophil elastase^14^. A similar time-dependent cleavage pattern was obtained with purified *P. aeruginosa* elastase (Fig. 1b).

Combined analysis using SDS–polyacrylamide gel electrophoresis (PAGE) of thrombin digested with *P. aeruginosa* elastase (Fig. 1c), N-terminal sequencing of the bands (Fig. 1c), high performance liquid chromatography (HPLC) of thrombin digested with elastase (Fig. 1d), and LC–MS/MS with fractions containing C-terminal thrombin peptides (Fig. 1e) identified the peptide FYT**HVFRLKKWIQKVIDQFGE** (FYT21). Interestingly, FYT21 has the same positive charge as the three amino acids shorter HVF18 (shown in bold letters in the above sequence). Figure 1f shows 2D and 3D representations of thrombin with the cleavage sites of *P. aeruginosa* elastase being coloured. The sequences TFGSG and IVEGS represent the N termini of the light and heavy chains, respectively. Notably, all cleavage sites are located in exposed regions of the thrombin molecule, except for the N-terminal cleavage site of the FYT21 sequence.

### FYT21 inhibits NFκB/AP-1 activation

To investigate possible actions of FYT21, the peptide was synthesized and compared with HVF18 using various assays. As a prelude to further analyses on eukaryotic cells, haemolysis assays were performed using either 25% whole blood or 0.5% erythrocyte solution (the latter representing the conditions in cell cultures). Whereas FYT21 did not significantly lyse erythrocytes in whole blood (Supplementary Fig. 1a), more than 10% lysis was observed in the 0.5% solution when using 20 μM or more (Supplementary Fig. 1b). HVF18 did not induce haemolysis (Supplementary Fig. 1c). Analysis of FYT21-induced LDH release by THP1 cells confirmed the above concentrations (Supplementary Fig. 1d). Based upon these results, FYT21 was used at concentrations up to 10 μM and HVF18 up to 40 μM.

**Figure 2:**
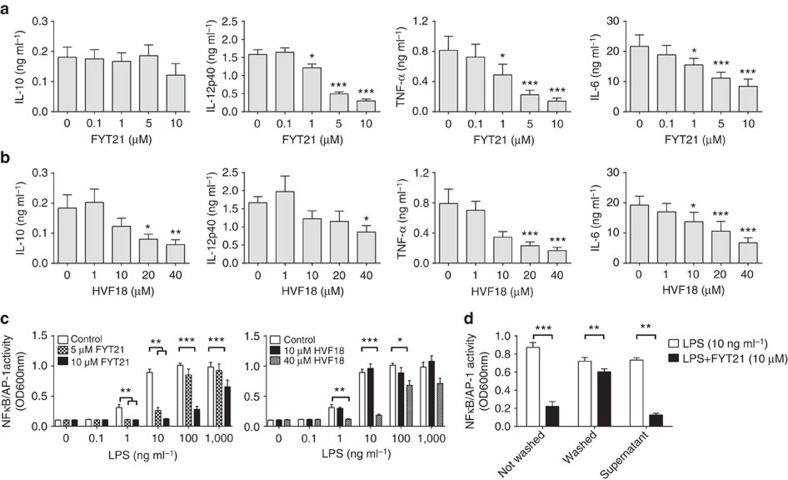
FYT21 reduces pro-inflammatory cytokine release by inhibiting NFκB/AP-1 activation. Whole blood (25%) was incubated overnight at 37 °C with FYT21 (**a**) or HVF18 (**b**) and 100 ng ml^−1^ of LPS, and supernatants were analysed by ELISA. (**c**) NFκB/AP-1 activity of THP1 reporter cells incubated overnight with a range of LPS with or without FYT21 or HVF18. (**d**) Cells were incubated with 10 μM FYT21 for 1 h, followed by adding LPS directly to the cells (Not Washed) or washing the cells first to remove unbound peptide. After centrifugation of the latter, the cells were suspended in fresh medium containing LPS (Washed), whereas the supernatants containing unbound peptide were transferred to naive cells followed by stimulation with LPS (Supernatant). Results are means±s.e.m. of 3–9 experiments. Values are significantly (*P<0.05, **P<0.005 and ***P<0.0005) different from the controls as analysed using a one-way ANOVA with a Dunnett's multiple comparisons test (**a**–**c**) or a paired *t*-test (**d**).

To investigate the effect of FYT21 on pro-inflammatory responses, 25% fresh venous human blood was incubated with FYT21 or HVF18 and simultaneously stimulated with LPS for 20 h. We found that FYT21 efficiently reduced TNF-α, IL-12p40 and IL-6 levels (Fig. 2a), whereas four times higher concentrations were needed for reduction of these cytokines with HVF18 (Fig. 2b). These results can be explained by our observation that both peptides dose dependently inhibited NFκB/AP-1 activation in response to various concentrations of LPS (Fig. 2c). Moreover, inhibition was increased by preincubation of the cells with FYT21 or HVF18 for 1 h before stimulation with LPS, but reduced when the cells were first stimulated with LPS for 1 h and then incubated with the peptides (Supplementary Fig. 1e,f). To investigate whether FYT21 exerts its effect by scavenging of LPS in the medium or by direct cell interactions, THP1 cells were incubated for 1 h with FYT21, washed with warm medium to remove unbound peptide, and then stimulated with LPS. As shown in Fig. 2d, removing unbound peptide still resulted in decreased NFκB/AP-1 activation by LPS, indicating that FYT21 binds to the cells. However, the effect was not as substantial as in conditions were unbound peptide remained present. Furthermore, the removed unbound peptide was capable of inhibiting the NFκB/AP-1 response when the FYT21-containing medium was transferred to naive cells followed immediately by LPS stimulation. Together, these results indicate that the peptide binds both LPS and eukaryotic cells.

### FYT21 binds to LPS and cell membranes

**Figure 3:**
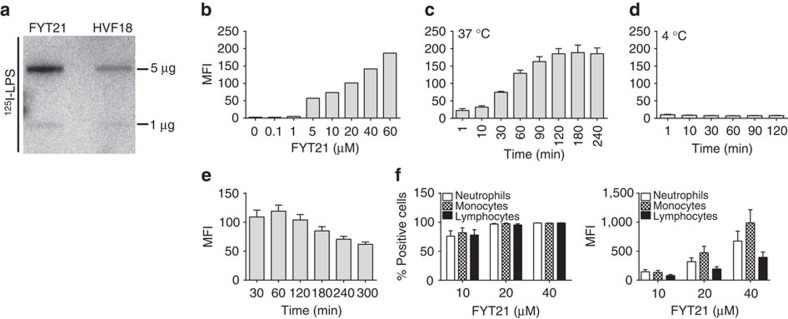
Binding of FYT21 to LPS and cell surfaces. (**a**) Binding of ^125^I-labelled LPS to FYT21 and HVF18 using a slot blot assay. (**b**) Binding of a range of TAMRA-labelled FYT21 to THP1 cells after 30 min incubation at 37 °C or (**c**) binding of 10 μM FYT21 after various incubation times at 37 °C or (**d**) 4 °C. (**e**) Cells were incubated with 10 μM FYT21 for 30 min, washed and the reduction in cell binding was followed for 5 h. (**f**) The percentage of FYT21 positive cells and the median fluorescence intensity (MFI) after incubation of whole blood with TAMRA-labelled FYT21. Results are means±s.e.m. of 3–8 experiments.

To assess the interaction between FYT21 and LPS, the peptides were blotted on a membrane and incubated with radiolabelled LPS. We found that both FYT21 and HVF18 bound to LPS (Fig. 3a). To investigate the interaction between FYT21 and monocytes, THP1 cells were incubated with TAMRA-labelled FYT21 and binding was measured using flow cytometry. The results showed that FYT21 dose (Fig. 3b) and time (Fig. 3c) dependently bound to THP1 cells at 37 °C, whereas no binding was observed at 4 °C (Fig. 3d). Furthermore, we found that the amount of peptide associated with the cells remained constant for at least 2 h after removal of unbound peptide (Fig. 3e). Binding of 10 μM FYT21 to the cell membrane was twice as high (median fluorescence intensity (MFI) of 108±9.3 versus 58.3±6.8) in the absence of 10% heat-inactivated fetal bovine serum (Supplementary Fig. 2a,b), indicating that serum components may bind to the peptide as well. The presence of LPS did not significantly alter the binding (Supplementary Fig. 2a,b). Notably, control experiments showed that 10 μM of TAMRA-labelled peptide was capable of significantly inhibiting NFκB/AP-1 activation from 1.03±0.06 to 0.16±0.13 (*n*=3), indicating that the label did not interfere with the peptide's activity.

To further investigate cell binding, THP1 cells were treated with trypsin or phosphatidylinositol-specific phospholipase C. The results showed that trypsin dose dependently decreased the binding of FYT21 with maximally 55% when using 10 μg ml^−1^, whereas phospholipase C (1 U ml^−1^) did not alter the binding (Supplementary Fig. 2c,d). CD14 expression was decreased with 35% after phospholipase C treatment, indicating that FYT21 binding to monocytes is not primarily dependent on the amount of cell-associated CD14.

To investigate whether peptide binding is monocyte specific, whole blood was incubated with TAMRA-FYT21 for 20 min and binding to cells was assessed. We found an equal percentage of peptide-containing monocytes, neutrophils and lymphocytes, although the MFI for monocytes was highest (Fig. 3f). Moreover, FYT21 also bound to platelets (Supplementary Fig. 2e). Together, these results suggest that FYT21 binds to a common cell membrane structure, although cell-specific receptor interactions cannot be excluded.

### FYT21 inhibits TLR4 activation by LPS

**Figure 4:**
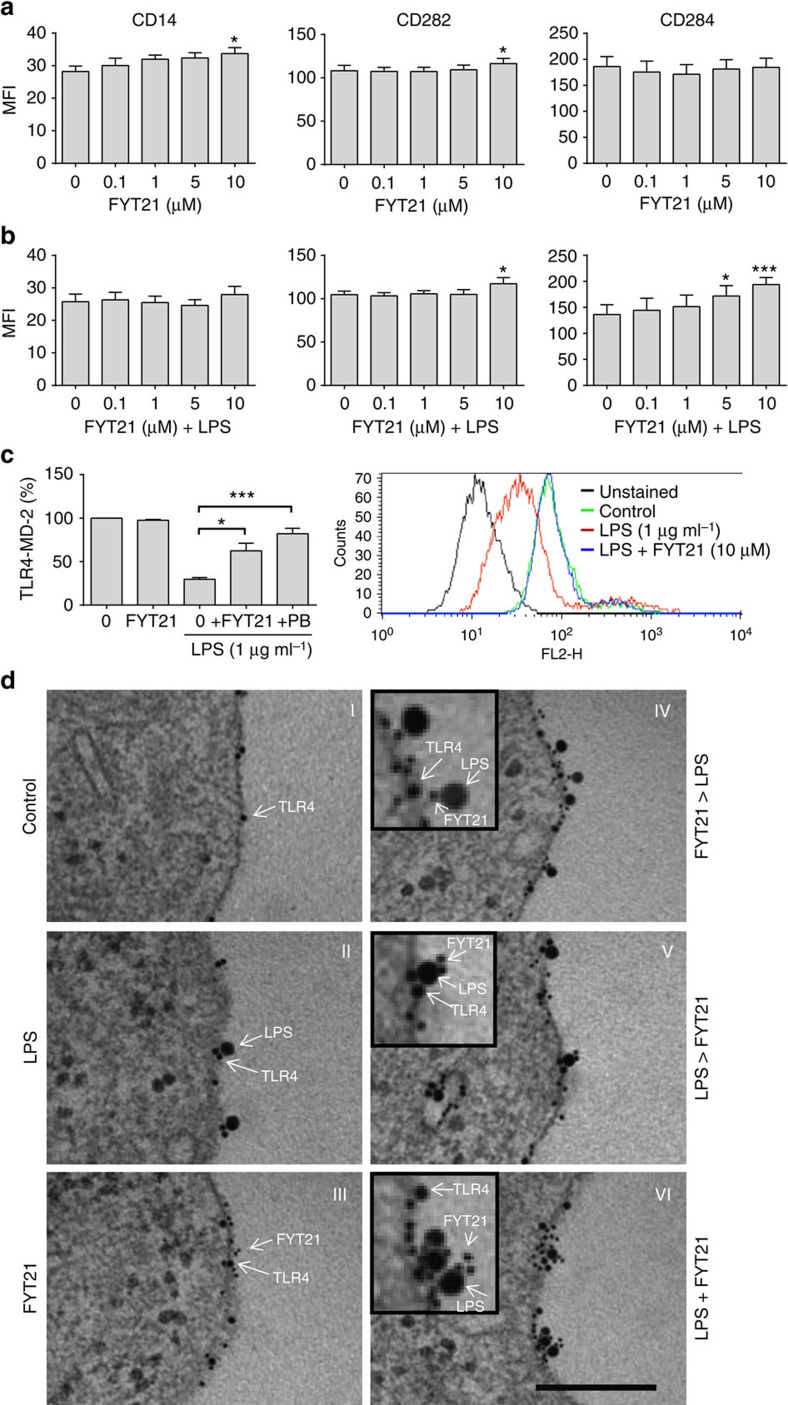
FYT21 inhibits TLR4 dimerization induced by LPS. THP1 cells were incubated o/n with a range of FYT21 without (**a**) or with (**b**) 100 ng ml^−1^ of LPS, and the expression of CD14, CD282 (TLR2) and CD284 (TLR4) was measured on a FACSCalibur. Results, expressed as mean fluorescence intensity (MFI), are means±s.e.m. of eight experiments. (**c**) The expression of monomeric TLR4 on RAW-Blue cells, as compared with dimerized/LPS-bound TLR4 (*n*=4), using an antibody that only binds to the monomeric form. A representative FACS histogram is shown to the right. Results of control samples were set at 100%. The effect of 10 μM FYT21 or 50 μg ml^−1^ of polymyxin B on LPS-induced TLR4 activation are expressed as a percentage relative to the control. Results are means±s.e.m. of four experiments. Values are significantly (*P<0.05 and ***P<0.0005) different from the controls as analysed using a one-way ANOVA with a Dunnett's multiple comparisons test. (**d**) Cells were incubated with LPS and/or FYT21 for 30–60 min followed by processing and Au-labelling with antibodies against TLR4, LPS and FYT21: (I) monomeric TLR4, (II) LPS-induced dimerization of TLR4, (III) binding of FYT21 to the membrane, (IV) the interaction between FYT21, LPS and TLR4 when the cells were preincubated for 30 min with FYT21 or (V) LPS, or when added simultaneously (VI; scale bar, 200 nm).

To investigate whether FYT21 influences the expression of LPS-binding receptors on the cell membrane, THP1 cells were incubated overnight with FYT21 at various concentrations in the presence and absence of 100 ng ml^−1^ of LPS, washed, stained with antibodies against TLR2 (CD282), TLR4 (CD284) and CD14, and finally receptor expression was measured on a FACSCalibur. The results showed that FYT21 did not affect the expression of TLR4 in the absence of LPS whereas the expression of CD14 and TLR2 was slightly enhanced (Fig. 4a). In the presence of LPS, TLR2 and TLR4 expression were significantly increased by FYT21 (Fig. 4b). Interestingly, LPS itself decreased the TLR4 expression, as compared with non-stimulated cells (described previously as LPS tolerance^21^), whereas FYT21 attenuated this decrease, indicating that the receptors did not get activated. Using an antibody that specifically recognizes TLR4/MD-2 complexes that are not bound to LPS, we confirmed with RAW macrophages that LPS loses its ability to bind and activate TLR4 complexes in the presence of FYT21 (Fig. 4c). LPS (1 μg ml^−1^) decreased the MFI to 29.3±2.1%, as compared with non-stimulated cells (100%), while the addition of 10 μM FYT21 increased the MFI to 67.5±8.7%. FYT21 alone did not alter the MFI values. In agreement with the results described for NFκB/AP-1 activation, stimulating the cells with LPS for 30 min before addition of the peptide did not induce a significant difference compared with LPS alone (Supplementary Fig. 3a). In contrast, addition of FYT21 30 min before stimulation with LPS recovered the MFI to 71.8±3.8% (Supplementary Fig. 3b), while preincubation of peptide and LPS further recovered the MFI to 77±9.9% (Supplementary Fig. 3c). The known LPS-binder polymyxin B (50 μg ml^−1^) was used as a control and showed a similar action as FYT21.

To visualize this interaction, THP1 cells were incubated with LPS, 10 μM FYT21 or combinations hereof for 30–60 min, and subsequently cells were fixed and processed for electron microscopy. Using antibodies against TLR4, LPS and FYT21, we observed monomers of TLR4 in the absence (Fig. 4d-I), and dimers in the presence of LPS (Fig. 4d-II), while FYT21 bound the membrane but did not induce TLR4 dimerization (Fig. 4d-III). Preincubating the cells for 30 min with FYT21, before addition of LPS, inhibited dimerization (Fig. 4d-IV), whereas FYT21 could not prevent dimerization and internalization when the cells were pretreated with LPS, although the peptide did bind to the LPS–TLR4 complex (Fig. 4d-V). Finally, when LPS and FYT21 were added simultaneously to the cells LPS primarily bound to the peptide (Fig. 4d-VI). Taken together, the results from these *in vitro* studies show that FYT21 binds to LPS in culture medium and on cell membranes thereby scavenging LPS away from its receptor thus preventing receptor activation.

### Clinical relevance of FYT21's activity

**Figure 5:**
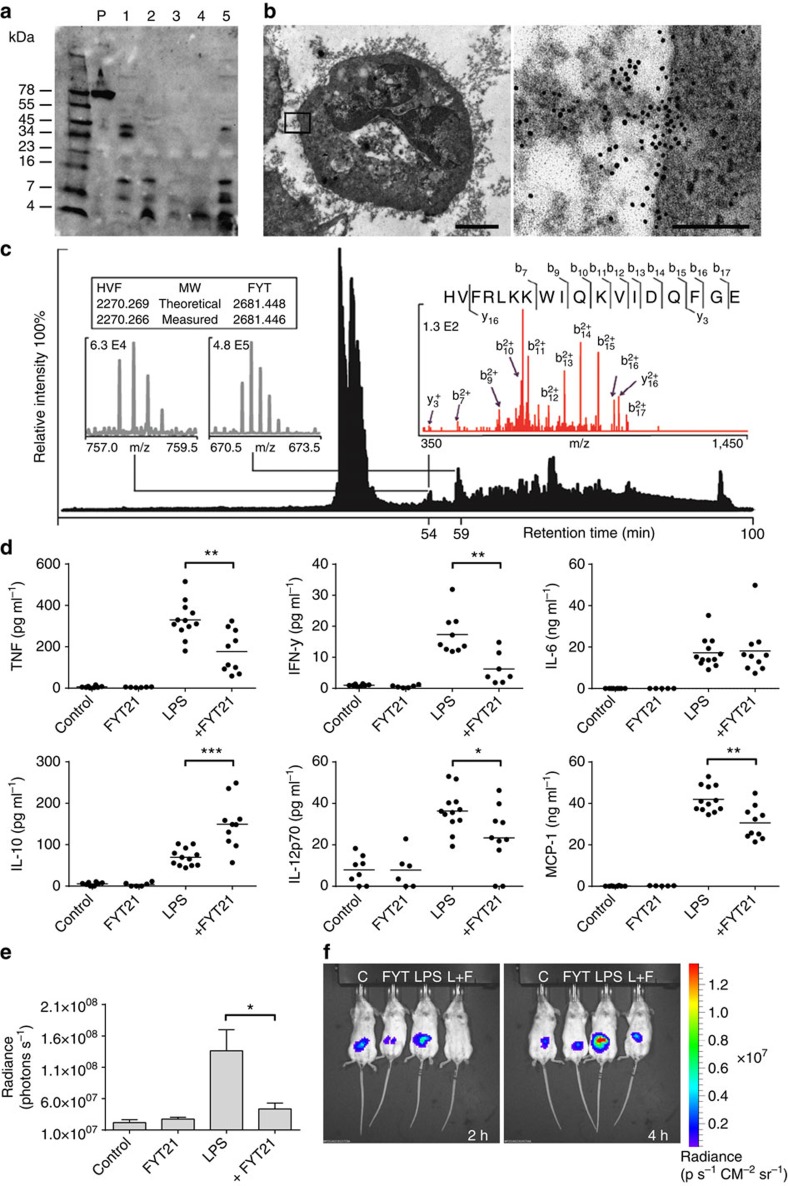
*In vivo* relevance of FYT21. (**a**) Wound fluids from non-healing leg ulcers (numbers 1–5) were analysed by western blotting using an antibody recognizing the C-terminal part of thrombin; plasma (p) is shown in lane 2. (**b**) An EM picture of a leukocyte, located in fibrin slough from an infected chronic wound, containing C-terminal epitopes of thrombin (black dots). The scale bars indicate 2 μm (left) and 100 nm (right). (**c**) Representative LC–MS/MS chromatogram of affinity purified wound dressing extract depicted in black; HVF18 eluted at 54 min and FYT21 at 59 min. The Orbitrap mass spectra of the triple-charged HVF18 peptide (*m*/*z* 757.7625) and the quadruple-charged FYT21 peptide (*m*/*z* 671.3670) enabled mass determination of both peptides (isotopic distribution depicted in grey). The assigned fragmentation pattern of HVF18 is given in red. (**d**) Cytokine analysis of blood collected from Balb/c mice (female 10–12 weeks), 4 h after i.p. injection with 1 mg kg^−1^ of LPS or PBS, which was followed after 30 min with injection of FYT21 (500 μg) or 10 mM Tris, pH 7.4. Control mice were injected twice with buffer. Results are means±s.e.m. of three independent experiments with 3–6 mice per group. (**e**) *In vivo* image of mice injected s.c. with the reactive oxygen species reporter probe L-012, 4 h after i.p. injection with 5 mg kg^−1^ of LPS and 500 μg FYT21 as described in **d**. Results are means±s.e.m. of three independent experiments with 3 mice per group. (**f**) A representative image of the four groups at 2 h and 4 h after injection: C, control; FYT, FYT21; L+F, LPS+FYT21. Values are significantly (*P<0.05, **P<0.005 and ***P<0.0005) different from the controls as analysed using a Mann–Whitney test.

To explore whether FYT21 is present during infection, human venous whole blood, incubated with 10% CM of 15159, or wound fluids from five patients with non-healing venous ulcers, chronically colonized with *P. aeruginosa*, were analysed by western blot using antibodies against the C-terminal part of thrombin. We detected bands similar in size to those observed after digestion of purified thrombin with *P. aeruginosa* elastase (Fig. 1b) in both whole blood (Supplementary Fig. 4a) and wound fluids (Fig. 5a), indicating that FYT21, or FYT21-related peptides, are indeed released during infection. Furthermore, electron microscopy of fibrin slough collected from such a wound showed the presence of thrombin-derived C-terminal epitopes both on a leukocyte cell membrane and the surrounding fibrin (Fig. 5b).

To further characterize these peptides, we incubated plasma after centrifugation of the blood samples from above (4 and 6 h time points) with VFR17 antibody-coated dynabeads and the bound peptides, comprising the C-terminal part of thrombin, were eluted and analysed using mass spectrometry. The results showed that both FYT21 and HVF18 were present in the whole-blood sample (Supplementary Fig. 4b), although the latter with less intensity, indicating that *P. aeruginosa* elastase and to a lesser extend neutrophil elastase contributed to the formation of C-terminal thrombin peptides.

Next, a wound fluid and a wound dressing extract (from different patients with *P. aeruginosa* infected non-healing ulcers) were incubated with VFR17 antibody-coated dynabeads and processed as described above. The results showed that FYT21 was indeed present in both patient samples; HVF18 was present in one of the patient samples, but with less intensity than FYT21. Figure 5c shows a representative LC–MS/MS chromatogram of the wound dressing extract (depicted in black). The reversed-phase gradient separated HVF18 and FYT21, eluting at 54 and 59 min, respectively. The Orbitrap mass spectra of the triple-charged HVF18 peptide (*m*/*z* 757.7625) and the quadruple-charged FYT21 peptide (*m*/*z* 671.3670) enabled mass determination of both peptides with high accuracy; the isotopic distribution of both peptides is depicted in grey. The identity of the peptides was further confirmed by database search of the MS/MS experiments. The assigned fragmentation pattern of HVF18 is shown in red, whereas the FYT21 pattern is already shown in Fig. 1e. Together, these results conclusively demonstrate that the C-terminal peptide FYT21 is present in wounds chronically colonised by *P. aeruginosa*.

Next we investigated whether dermatan sulphate, a commonly found glycosaminoglycan in wounds^22^, could inhibit FYT21's actions. We found that concentrations of 50 μg ml^−1^ and higher significantly inhibited cell binding and NFκB/AP-1 inhibitory activity of the peptide (Supplementary Fig. 5a,b). However, as the mean concentration of glycosaminoglycans in wound fluids is around 20 μg ml^−1^ (0–40 μg ml^−1^)^23^, we assume that under physiological conditions, the immunomodulatory activity of FYT21 will not be affected significantly.

Finally, we explored the *in vivo* activity of FYT21. Balb/c mice were injected intraperitoneally (i.p.) with 1 mg kg^−1^ of LPS, followed by injection of 500 μg of FYT21 after 30 min, and subsequent blood collection for cytokine analysis after another 210 min. The 4 h time frame was based on initial time-response experiments (Supplementary Fig. 6a), where some cytokine levels were highest 2 h after injection with LPS whereas others were higher after 6 h. The results showed that FYT21 significantly reduced TNF, MCP-1, IL-12p70 and IFN-γ release, whereas the levels of the anti-inflammatory cytokine IL-10 were increased (Fig. 5d), confirming the *in vitro* observed inhibition of pro-inflammatory responses. FYT21 itself did not induce alterations in cytokine release. In addition, FACS analyses of intraperitoneal cells, collected after injection of mice with TAMRA-FYT21, showed binding of the peptide to the different cell populations, both in the presence and absence of LPS (Supplementary Fig. 6b). To further explore the *in vivo* actions of FYT21, we used the luminescent reactive oxygen species probe L-012. In agreement with a previous report^24^, we found that subcutaneous (s.c.) injection of the probe, as compared to intraperitoneal or intravenous injection, gave the most stable signal (Supplementary Fig. 6c). Notably, 1 mg kg^−1^ of LPS did not give reproducible results (Supplementary Fig. 6d) but 5 mg kg^−1^ did (Supplementary Fig. 6e). The signal strength induced by 5 mg kg^−1^ of LPS was relatively steady 10 to 18 min after injection of the probe. Based upon these results, 12 min was used to test the effect of FYT21. Using the same experimental set up as used for the cytokine analysis, the results showed that FYT21 was capable of reducing ROS production induced with 5 mg kg^−1^ of LPS (Fig. 5e,f). Notably, already 2 h after the start of the experiment FYT21 showed reduced ROS activity, as compared with LPS (Fig. 5f). Taken together, these results show that FYT21 is released in non-healing *P. aeruginosa* infected wounds and that this peptide can repress inflammation *in vivo* by decreasing the release of pro-inflammatory cytokines and reactive oxygen species.

### FYT21 inhibits cell activation by various TLR agonists

**Figure 6:**
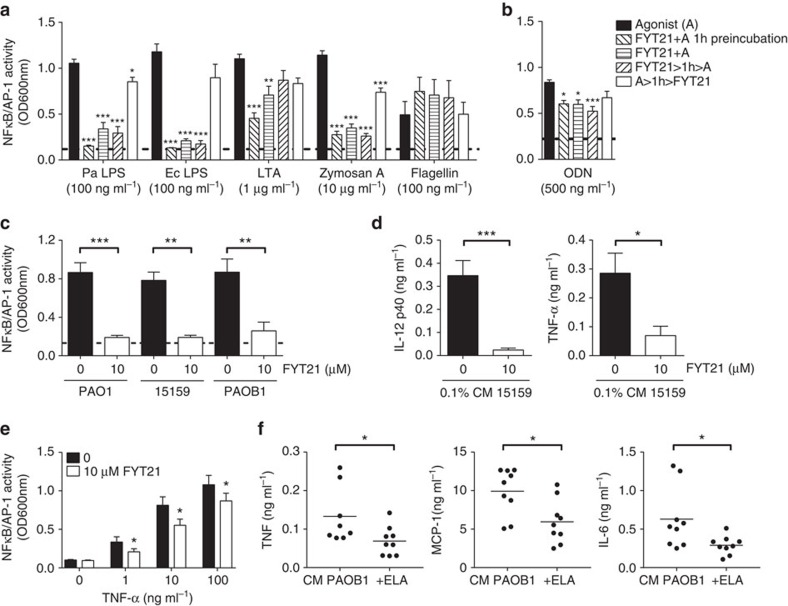
FYT21 inhibits cell activation by various TLR agonists. NFkB/AP-1 activity of THP1 reporter cells (**a**) or RAW reporter cells (**b**) stimulated with the TLR2/4 agonists LPS of *P. aeruginosa* (Pa) and *E. coli* (Ec), the TLR2 agonists LTA and zymosan, the TLR5 agonist flagellin, or the TLR9 agonist ODN 1 h before (A>1 h>FYT21) or after (FYT21>1 h>A) incubation with 10 μM FYT21, together with FYT21 after 1 h preincubation (FYT21+A 1 h preincubation) or FYT21 and agonist were mixed in the cell culture well (FYT21+A). (**c**) THP1 reporter cells were preincubated for 1 h with 10 μM FYT21 followed by stimulation with conditioned medium (CM) of three strains of *P. aeruginosa*. (**d**) Cytokine analysis of whole blood stimulated similarly as **c** with CM of the clinical strain 15159. (**e**) Effect of FYT21 on stimulation with recombinant TNF-α. Results are means±s.e.m. of 4–9 experiments. (**f**) Cytokine analysis of blood collected from Balb/c mice (female 10–12 weeks), 4 h after s.c. injection with 100 μl CM of PAOB1 supplemented with 3 units *P. aeruginosa* elastase or 10 mM Tris buffer. Results are means±s.e.m. of three independent experiments with 3 mice per group. Values are significantly (*P<0.05, **P<0.005 and ***P<0.0005) different from the controls as analysed using a one-way ANOVA with a Dunnett's multiple comparisons test (**a**,**b**), a paired *t*-test (**c**–**e**) or a Mann–Whitney test (**f**).

To determine whether inhibition of pro-inflammation by FYT21 is specific for stimulation with *P. aeruginosa* LPS, we assessed the effect of FYT21 on NFκB/AP-1 activation by various pathogen-associated molecular patterns. The results showed that FYT21 inhibited cell activation induced by different types of LPS and zymosan A, and to a lesser extent that induced by LTA (Fig. 6a), indicating that inhibition by FYT21 is not LPS or receptor specific. The response towards flagellin was not reduced by the peptide. In agreement with the above *in vitro* findings for LPS, preincubation of peptide and stimuli for 1 h induced the largest inhibition, suggesting the presence of a peptide–agonist interaction. In addition, FYT21 inhibited ODN-induced NFκB/AP-1 activation of RAW reporter cells (Fig. 6b). In agreement with the above data, FYT21 inhibited NFκB/AP-1 activation by 0.1% bacterial-derived conditioned medium (Fig. 6c) as well as the production of TNF-α and IL-12p40 in human whole blood (Fig. 6d). Finally, we found that FYT21 its actions are not limited to PAMPs as the peptide was also capable of inhibiting NFκB/AP-1 activation by recombinant TNF-α (1–100 ng ml^−1^) with ∼30% (Fig. 6e).

As FYT21 is released by *P. aeruginosa* elastase, we finally investigated whether the purified enzyme could induce inhibition of pro-inflammatory activities *in vivo*. For this purpose Balb/c mice were injected s.c. with 100 μl of conditioned medium from the elastase-deficient PAOB1 strain supplemented with 3 units of *P. aeruginosa* elastase or 10 mM Tris as a control, and subsequently blood was collected for cytokine analysis after 4 h. The results showed that elastase significantly reduced TNF, MCP-1 and IL-6 levels (Fig. 6f) whereas IL-12p70 and IFN-γ were below detection limit (2 pg ml^−1^). The level of anti-inflammatory cytokine IL-10 was not significantly affected (mean±s.e.m. of 21.46±2.25 versus 18.07±2.35 pg ml^−1^).

Taken together, FYT21 inhibits stimulation of THP1 cells and whole blood by various TLR agonists, bacterial conditioned medium containing a large variety of virulence factors and PAMPs, and the pro-inflammatory cytokine TNF-α. Moreover, *P. aeruginosa* elastase reduced inflammatory cytokine production *in vivo*, showing a proof of principle that the bacterial enzyme indeed reduces pro-inflammatory responses under physiological conditions.

### Effect of FYT21 on bacterial survival

**Figure 7:**
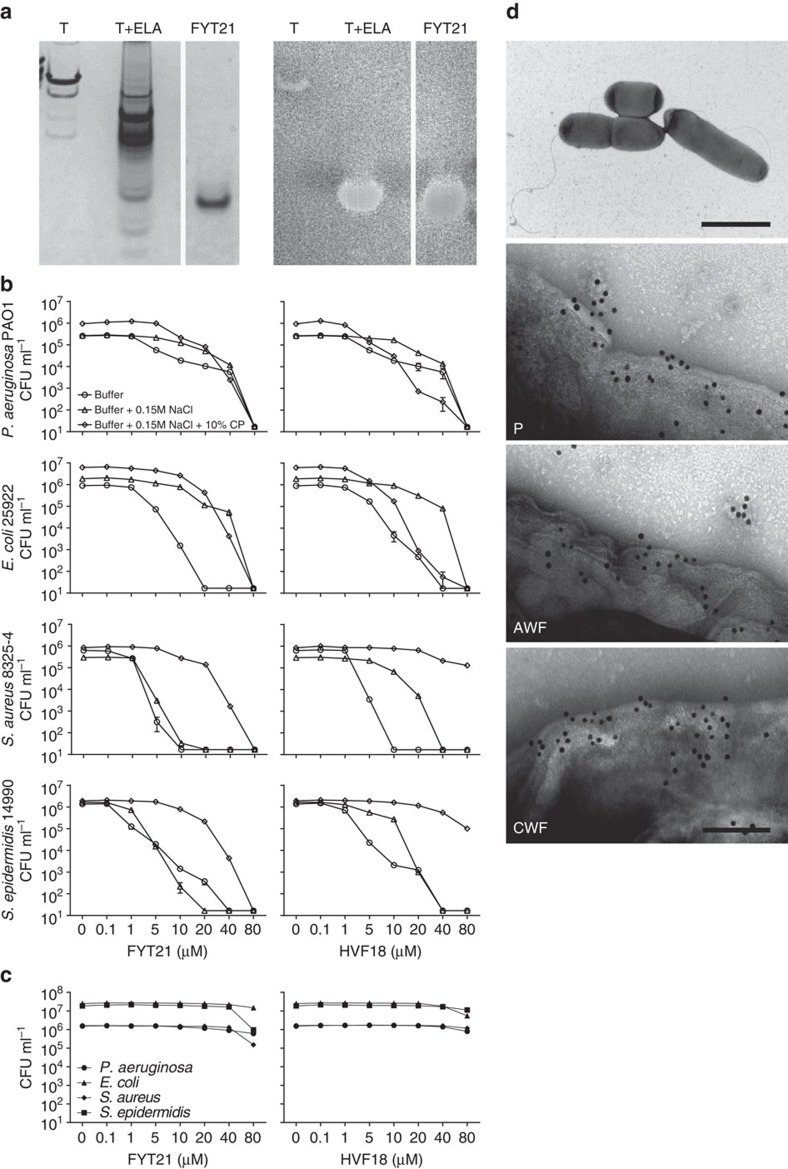
The interaction between bacteria and FYT21. (**a**) Thrombin was digested with *P. aeruginosa* elastase and the resulting material was used in a bacterial overlay assay to assess bactericidal activity against *E. coli*. FYT21 was run in parallel; the lane is from the same gel but aligned for clarity. (**b**) Antimicrobial activity of FYT21 or HVF18 against various Gram-negative and Gram-positive bacteria in exponential phase in 10 mM Tris buffer containing 5 mM glucose and/or supplemented with 0.15 M NaCl or 0.15 M NaCl and 10% citrate plasma (cp), or (**c**) in 25% human whole blood and incubated for 2 h with a range of FYT21 or HVF18. Results are means±s.e.m. of 4 experiments. (**d**) *P. aeruginosa* 15159 was incubated with plasma (P), acute wound fluid (AWF) or chronic wound fluid (CWF) for 30 min and binding of thrombin-derived C-terminal epitopes to the bacterial membrane was visualized using electron microscopy (scale bars, 2 μm (overview image) and 100 nm).

As HVF18 is reported to exert antimicrobial activity *in vitro*, we investigated whether thrombin degradation by *P. aeruginosa* elastase also induces antimicrobial activity. Using a bacterial overlay assay, we indeed found that FYT21 shows antimicrobial activity against *E. coli* (Fig. 7a). Next we investigated the physiological relevance of the observed antimicrobial activity using various buffer conditions. Although both FYT21 and HVF18 were antimicrobial against Gram-positive and Gram-negative bacteria in Tris buffer supplemented with 5 mM glucose (Fig. 7b), the addition of 0.15 M NaCl attenuated this effect in most conditions, as did further addition of 10% citrated plasma. Moreover, antimicrobial activity of the peptides (at doses up to 80 μM) in 25% whole blood was abrogated (Fig. 7c). Interestingly, electron microscopy showed that thrombin-derived C-terminal peptides bind to bacterial membranes when incubated in plasma, acute wound fluid or chronic wound fluid (Fig. 7d). Taken together, these results underscore the importance of FYT21's immunomodulatory effects vis-à-vis its direct bactericidal effects *in vivo*, assuming physiological concentrations as those reported for thrombin (∼1.4 μM)^25^.

## Discussion

*P. aeruginosa* is a Gram-negative opportunistic pathogen known for its immune evasive abilities. In the present work, we describe a novel concept, where bacteria mimic an endogenous anti-inflammatory peptide-based mechanism to suppress host responses. Our main finding is that *P. aeruginosa* elastase cleaves the coagulation protein thrombin, thereby releasing a C-terminal-derived peptide that binds to pathogen-associated molecular patterns such as LPS, thus preventing cell activation and subsequent pro-inflammatory responses. This conclusion is based on the following observations. First of all, using two independent methods we identified a 21 amino acid long cationic C-terminal peptide, designated FYT21, released after digestion of thrombin with *P. aeruginosa* elastase. Our results showed that synthetically produced FYT21 was capable of inhibiting pro-inflammatory cytokine release and NFκB/AP-1 activation in response to LPS, but also to other *P. aeruginosa*-associated PAMPs (that is, ODNs) and non-associated PAMPs (that is, *E. coli* LPS, zymosan, LTA), as well as the cytokine TNF-α. The response towards flagellin was not inhibited. However, alkaline protease produced by *P. aeruginosa* can degrade monomeric flagellin^26^ thereby preventing recognition via TLR5. In agreement, FYT21 also inhibited NFκB/AP-1 activation and cytokine release in response to conditioned medium from several *P. aeruginosa* strains, which contains many different PAMPS and virulence factors including flagellin. The *in vitro* activity of FYT21 was confirmed *in vivo* by cytokine analyses and employing *in vivo* imaging of ROS production. Moreover, *P. aeruginosa* elastase itself reduced inflammatory cytokine production *in vivo*, showing a proof of principle that this enzyme indeed reduces pro-inflammatory responses under physiological conditions. Finally, we show the mode of action of this peptide during LPS stimulation. We discovered that FYT21 binds to LPS in solution as well as on cell membranes, thereby acting as a scavenger preventing TLR4 dimerization and subsequent cell activation.

Of particular clinical relevance is the finding that FYT21 is present in infected chronic wounds and is bound to leukocytes and fibrin. Although the concentrations of thrombin-derived peptides in chronic wounds are unknown, based on the concentration of prothrombin in plasma (1.4 μM (ref. 25)) and the fact that large amounts of exudate are deposited in chronic wounds, we consider the concentrations of peptide used in our studies of physiological relevance. Our *in vivo* data further underscore the anti-inflammatory role of FYT21. *In vitro* a FYT:LPS ratio of around 25:1 (w/w) was required to significantly reduce the LPS-induced pro-inflammatory cytokine release. The same ratio was successfully used *in vivo* but LPS was injected 30 min before FYT21 instead of given simultaneously, while the former method was shown to be much less effective in inhibiting NFκB/AP-1 activity *in vitro*. In this context, inhibition of TLR agonist-induced NFκB/AP-1 activation was paramount when agonist and FYT21 were preincubated. Since all affected agonists are negatively charged, it may be hypothesized that electrostatic interactions seem to play a pivotal role in their interaction with the cationic FYT21. In agreement, FYT21 was capable of binding all tested cell types in whole blood and intraperitoneal washes, as well as bacteria indicating similar interactions with negatively charged cell membranes. However, monocytes had a significant higher amount of bound peptide than the similar sized neutrophils. Therefore, we cannot exclude additional specific membrane or protein interactions.

From a structural viewpoint, it is interesting that FYT21's structure is comparable to that of many helical antimicrobial peptides/HDPs and cell-penetrating peptides^27^,^28^. Furthermore, previous structure-activity work, aimed at defining host defence epitopes of a prototypic 25mer peptide (GKY25) of the C-terminal region of thrombin, shown to execute anti-inflammatory effects *in vitro* and *in vivo*^14^,^15^, demonstrated that a central ‘core' containing a conserved WI motif surrounded by cationic residues is important for the peptide's antimicrobial activity^29^. Furthermore, the observation that an internal fragment of GKY25 of 12 amino acids (VFRLKKWIQKVI) bound LPS but showed no anti-endotoxic effects upon macrophage stimulation, indicated length-dependent interactions influenced by helix formation of these peptides. In agreement with these results, we observed that the shorter HVF18 was less potent than FYT21. From a host perspective, these results thus demonstrate that host defence sequences are encoded in larger proteins, and can be released upon proteolytic activation. It is interesting that *P. aeruginosa* by its production of elastase exploits this mechanism, releasing peptides which contain such biophysically governed host defence motifs. As the formation of an antimicrobial peptide would clearly be disadvantageous for *P. aeruginosa*, we investigated the importance of FYT21's bactericidal effects *in vivo*. Indeed while FYT21 was capable of killing *P. aeruginosa* and several other types of bacteria in buffer conditions *in vitro*, mimicking physiological conditions using plasma reduced this activity, while the presence of whole blood abrogated the bactericidal effects, underscoring the importance of the immunomodulatory effects for this peptide's action *in vivo.*

From an evolutionary perspective, it is not surprising that bacteria have evolved ways of mimicking the formation of HDPs. Clearly, *P. aeruginosa* produces many different proteases that are capable of cleaving numerous host proteins, thereby making it likely that other peptides, with novel functions may be released as well. It may be argued that *P. aeruginosa*, believed to be mainly present in soil, water, decomposing organic materials but also found in the intestinal flora^30^, obviously does not have a need for evolving enzymes that lead to release of bioactive peptides in a human host. However, recent evidence, demonstrates that *P. aeruginosa* is also highly abundant in human as well as murine skin^31^,^32^, indicating that the pool of bacteria interacting with humans and other animal species is bigger than considered previously. This observation, together with the fact that the C terminus of thrombin is highly conserved during evolution^14^,^33^ (Supplementary Fig. 7), opens up possibilities for the presence of evolutionary pressure based on host–pathogen interactions and involving manipulation of host defence. In line with this reasoning are also recent findings suggesting that *P. aeruginosa* undergoes a significant convergent evolution in cystic fibrosis patients^34^. *P. aeruginosa* elastase belongs to a superfamily of zinc-containing metallopeptidases of the M4 (thermolysin) class, also secreted from bacteria such as *Enterococcus faecalis* (coccolysin) and *Staphylococcus aureus* (aureolysin)^35^, bacteria very common in chronic wounds^36^. Hence, it is tempting to speculate that formation of anti-inflammatory HDPs is not specific for *P. aeruginosa*, but may constitute a more common mechanism by which bacteria evade and modulate host immune responses.

## Methods

### Ethics statement

The use of human wound material was approved by the Ethics Committee at Lund University (LU 708-01 and LU 509-01). Written informed consent was obtained from the participants. The animal models were approved by the Laboratory Animal Ethics Committee of Malmö/Lund (permit numbers M252-11 and M226-12) and experiments were conducted according to the guidelines of the Swedish Animal Welfare Act SFS 1988:534.

### Peptides and proteins

Prothrombin and human α-thrombin were obtained from Innovative Research, Inc, USA. The thrombin-derived C-terminal peptides HVF18 (HVFRLKKWIQKVIDQFGE), FYT21 (FYTHVFRLKKWIQKVIDQFGE) and TAMRA-labelled (at the N terminus) FYT21 were synthesized by Biopeptide Co, Inc., USA. The purity and molecular weight of these peptides were confirmed by MS analysis.

*P. aeruginosa* elastase was a kind gift of Dr H. Maeda, Kumamoto University, Japan. One unit of elastase is defined as the amount of enzyme capable of digesting 1 mg azocasein (Sigma-Aldrich, USA) per hour at 37 °C. In all assays, digestion was performed using 4 milli-units (mU) of elastase per μg thrombin.

### Biological materials

Wound fluids from patients with non-healing venous ulcers chronically colonised with *P. aeruginosa* were collected under a Tegaderm dressing for 2 h as described previously^37^. Wound fluids were centrifuged and stored at −20 °C. Wound dressing extracts of such patients were obtained using wound dressing material (polyurethane) that had been placed on the wound for 3–4 days. A length of 1 cm^2^ of dressing was transferred to a 2 ml tube containing 300 μl 10 mM Tris buffer (pH 7.4) and 200 μl of 1.4 mm ceramic beads (MO BIO Laboratories, Inc, USA) and the sample was homogenized in four repeats of 15 s at 7,000 r.p.m. using a MagNA Lyser (Roche Diagnostics GmbH, Germany). Next, the dressing extract was collected after centrifugation for 5 min at 4,000*g* followed by heat treatment for 10 min at 95 °C and subsequent storage at 4 °C. Fibrin slough was collected using a sterile spatula and fixed immediately for electron microscopy.

### Bacterial strains and growth conditions

*P. aeruginosa* PAO1 and PAOB1 (ref. 38) (kindly provided by B. Iglewski) and clinical strains 15159, 3.1, 10.5 and 14.1, *Escherichia coli* 25922, *S. aureus* 8325-4 and *Staphylococcus epidermidis* 14990 were grown in Todd-Hewitt medium at 37 °C under vigorous shaking. To obtain CM, bacteria grown for 24 h were centrifuged at 5,000*g* for 15 min and the culture media were sterilized through a 0.22 μm filter.

### Acid-urea gel electrophoresis

Acid-urea gels (AU-PAGE; 12.5%) were prepared as described^39^. Ten microgram of human α-thrombin in 10 mM Tris buffer (pH 7.4) was incubated with *P. aeruginosa* elastase for 1 h at 37 °C and then mixed with sample buffer (9 M urea in 5% acetic acid) in a 2:1 ratio. Next, samples were transferred to the slots and gels were run in duplicate in 5% acetic acid for 90–120 min at 100 V using reversed polarity. Thereafter, one gel was stained with Coomassie Brilliant blue R250 (Imperial Chemical Industries PLC, Merck, Germany) whereas the other was used in the overlay assay.

### SDS-gel electrophoresis

Human α-thrombin (4–8 μg) in 10 mM Tris buffer (pH 7.4) was incubated with *P. aeruginosa* elastase or 10% CM for various time intervals at 37 °C. Next, samples were denatured at 95 °C for 5 min in 1 × reducing SDS sample buffer followed by separation on 10–20% Tris-Tricine mini gels in 1 × Tris-Tricine SDS running buffer for 75 min at 125 V. Gels and buffers were derived from Invitrogen (USA). Gels were stained with Coomassie Brilliant blue and patterns were visualized using a Gel Doc Imager (Bio-Rad Laboratories, USA).

### Western blot

After SDS-gel electrophoresis, gels were equilibrated in 1 × Tris-Glycine SDS transfer buffer (Invitrogen) for 5–10 min, assembled in transfer cassettes and placed in a Criterion blotter (Bio-Rad) all according to manufacturer's instructions; the transfer was performed at 100 V for 60 min. Subsequently, the nitrocellulose membrane (Hybond-C, Amersham BioSciences, UK) was rinsed in H_2_O, blocked (5% non-fat dry milk in PBS containing 0.05% Tween-20) for 60 min at RT while shaking, and incubated overnight at 2–8 °C with polyclonal rabbit anti-human antibodies against the C-terminal thrombin peptide VFR17 (VFRLKKWIQKVIDQFGE; Innovagen AB, Sweden). Next, the membrane was washed thrice in PBS supplemented with 0.05% Tween-20 and incubated with swine anti-rabbit immunoglobulin—HRP conjugated antibodies (DAKO A/S, Denmark) for 60 min at room temperature. Finally, the membrane was washed, developed with SuperSignal West Pico Chemiluminescent Substrate (Thermo Scientific, Denmark) for 5 min and visualized using a ChemiDoc XRS Imager (Bio-Rad).

### N-terminal sequencing

Human α-thrombin (30 μg) was digested with *P. aeruginosa* elastase for 1 h, separated on a 10–20% Tris-Tricine gel, blotted onto an Immobilon-pSQ transfer membrane (Millipore, USA) and stained with Coomassie Brilliant blue. The different peptide bands were marked out and sent for N-terminal sequencing to Karolinska Institute HEJ-Analyser (Sweden).

### HPLC

Human α-thrombin (300 μg) was digested with *P. aeruginosa* elastase for 1 h and then injected into a HPLC system (PerkinElmer Series 200; PerkinElmer, USA) equipped with a Vydac 218TP C18 (5 μm, 4.6 mm × 250 mm) reverse phase column (Grace Discovery Sciences, USA). The mobile phases consisted of (A) 0.05% trifluroacetic acid in H_2_O and (B) 0.05% trifluroacetic acid in acetonitrile. A 70 min linear gradient from 5 to 95% B was applied at a flow rate of 1 ml min^−1^. Next, 60 fractions of 1 ml were collected, freeze-dried, solubilized in 25 μl H_2_O and used in further experiments.

### Orbitrap mass spectrometry

Samples were acidified and analysed by online nanoflow liquid chromatography tandem mass spectrometry (LC−MS/MS). LC−MS/MS-experiments were performed on an Acclaim PepMap EASY-nLC system (Thermo Scientific) connected to a LTQ Orbitrap Velos Pro (Thermo Scientific) through a nanoelectrospray ion source. Peptides were loaded by an autosampler, using the intelligent flow control mode, onto an Acclaim PepMap EASY-column (2 cm, 75 μm inner diameter) packed with reversed-phase C18 5 μm particles (Thermo Scientific). The flow rate was reduced to maximum 300 nl min^−1^ and the peptides were separated on a PepMap EASY-column (10 cm long, 75 μm inner diameter) packed with reversed-phase C18 3 μm particles using a linear ACN gradient from 2 to 35% in 0.1% formic acid for 60 min followed by a linear increase to 98% ACN over 5 min. The LTQ Orbitrap Velos Pro instrument was operated in the data dependent mode to automatically switch between full scan MS and MS/MS acquisition. Survey full scan MS spectra (*m*/*z* 350−1,800) were acquired in the Orbitrap with 60,000 resolution after accumulation of ions to a 1 × 10^6^ target value based on predictive AGC from the previous full scan. Dynamic exclusion was set to 30 s and MS/MS acquisition was performed in FT mode with a resolution of 7,500. Typical targeted parameters included an isolation width of two bracketed around the *m*/*z* of interest, fragmentation time of 10 ms, normalized collision energy of 35, spray voltage of 2.0 kV, and capillary temperature at 275 °C. Raw data were processed by Mascot distiller and MGF files were created and searched against Swiss-Prot database (release of 03-march-2011, containing 515,203 entries) on an in-house Mascot database (version 2.3.02). No enzyme was used and oxidized methionine was searched as variable modifications.

### Detection of FYT21

Dynabeads M-280 sheep anti-rabbit IgG (Novex Life Technologies) were used according to manufacturer's instructions, In short, 100 μl beads in washing buffer and 100 μl VFR17 antibody were incubated overnight at 4 °C while rotating at 7 r.p.m. Next, the beads were washed, incubated for 1 h at room temperature while rotating at 7 r.p.m. with 100 μl plasma derived from whole blood incubated with 10% CM, chronic wound fluid or wound dressing extract, and washed again in washing buffer and subsequently in 10 mM Tris buffer (pH 7.4). Next, thrombin-derived C-terminal peptides were eluted off the beads in 2 steps of 40 μl each using 0.1 M citrate (pH 2.2). Analyses of the eluate was performed on the Orbitrap system described above using a linear ACN gradient from 5 to 25% in 0.1% formic acid for 60 min followed by a linear increase to 45% ACN over 30 min, and finally an increase to 100% over 5 min.

### Slot blot assay

Peptides were bound to nitrocellulose membranes pre-soaked in PBS. Next, membranes were blocked (2% w/v BSA in PBS, pH 7.4) for 1 h at room temperature followed by incubation with ^125^I-labelled LPS (5 × 10^5^ c.p.m. per ml)^40^ for 1 h. Membranes were washed three times 10 min in PBS and radioactivity was visualized using a Bas 2000 radio imaging system (Fuji, Japan).

### Overlay assay

Bacteria in mid-log phase were centrifuged at 2,000*g* for 10 min and washed with 10 mM Tris pH 7.4. Next, 4 × 10^6^ CFU were dispersed in 15 ml of underlay agarose gel consisting of 1% (w/v) low electroendosmosis type (EEO) agarose (Sigma-Aldrich), 0.03% (w/v) TSB and 0.02% (v/v) Tween-20 in 10 mM Tris HCl buffer pH 7.4 at 42 °C. Subsequently, the agar was poured into Petri dishes (144 mm in diameter) and solidified. Acid-urea gels were prepared as described above. Next, gels were washed thrice with 100 ml of sterile 10 mM Tris (pH 7.4) for 4 min and placed on top of the agar. After 3 h incubation at 37 °C, gels were removed and stained with Coomassie Brilliant blue. In parallel, 15 ml of overlay agar (6% TSB and 1% low-EEO agarose) was poured on top of the bacterial underlay. After overnight incubation at 37 °C, plates were fixed for 3 h in 5% acetic acid using gentle agitation followed by staining with Commassie brilliant blue.

### *In vitro* killing

Bacteria in mid-log phase were centrifuged at 3,000*g* for 10 min, washed twice in PBS and suspended in 10 mM Tris (pH 7.4) supplemented with 5 mM glucose, with or without 0.15 M NaCl and 10% human citrate plasma, or in RPMI-1640 (Gibco Life Technologies ltd, UK) to a concentration of 2 × 10^6^ c.f.u. per ml. Subsequently, 50 μl of the bacterial suspension was transferred to Eppendorf tubes containing a range of peptide (0–80 μM) in 50 μl of the same buffer or in equal parts of venous blood and RPMI-1640. After 2 h incubation, the number of surviving bacteria was determined microbiologically using serial dilutions of these suspensions plated in six-fold onto agar plates.

### Haemolysis assay

Fresh venous blood from healthy donors, collected in the presence of the anti-coagulant lepirudin (50 μg ml^−1^), was centrifuged at 250*g* and the pellet was washed three times with PBS. Next, the pellet was diluted 100 times and 100 μl of this solution was transferred to each well of a 96-wells plate containing 100 μl of sample in PBS. Alternatively, 50 μl of blood was transferred directly to wells containing 150 μl of sample in RPMI. After 1 h incubation at 37 °C and 5% CO_2_, the plate was centrifuged at 800*g*, 150 μl of each sample was transferred to a flat-bottom 96-wells plate and the absorbance at 450 nm was measured. Results are expressed as percentage of erythrocyte lysis compared to the positive control (2.5% Tween-20). Values below 10% are regarded as non-haemolytic.

### Whole-blood assay

Fresh venous blood from healthy donors, collected in the presence of lepuridin (50 μg ml^−1^), was mixed with 3 parts RPMI-1640—GlutaMAX-I (Gibco). Next, 1 ml of this mixture was transferred to each well of a 24-wells plate and incubated with a range of FYT21, HVF18 or, as a control, H_2_O and stimulated with 100 ng ml^−1^ of LPS (Sigma Chemical Co., USA), 0.1% CM of *P. aeruginosa* strain 15159 or no stimulus. After 18–22 h incubation at 37 °C and 5% CO_2_, supernatants were collected and stored at −70 °C.

### NFκB and AP-1 activity measurement

THP1-Xblue-CD14 reporter cells (InvivoGen, France) were cultured and stimulated following manufacturer's instructions. Cells were incubated with 0.1–10 μM FYT21 or 1–40 μM HVF18 and stimulated with 1–1,000 ng ml^−1^ of *P. aeruginosa* or *E. coli* LPS (Sigma), 1 μg ml^−1^ purified LTA (InvivoGen), 10 μg ml^−1^ zymosan A (Sigma), 100 ng ml^−1^ purified flagellin (InvivoGen), 1–100 ng ml^−1^ recombinant TNF-α (InvivoGen), 0.1% CM of various *P. aeruginosa* strains or no stimulus. In addition, cells and/or peptide were pretreated with 5–100 μg ml^−1^ dermatan sulphate (DS36)^41^. After 18–22 h incubation at 37 °C and 5% CO_2_, 20 μl of the supernatants were transferred to new 96-wells plates and 180 μl of QUANTI-Blue (InvivoGen) was added. Plates were incubated at 37 °C and the levels of secreted embryonic alkaline phosphatase (SEAP), which is controlled by the activation of transcription factors NFκB and AP-1, were measured after 1–2 h at an absorbance of 600 nm. To establish the effect of FYT21 on activation of NFκB/AP-1 via TLR9, RAW-Blue reporter cells (InvivoGen) were cultured according to manufacturer's instructions, incubated with 10 μM FYT21 and stimulated with 500 ng ml^−1^ ODN1826 (InvivoGen).

### LDH assay

THP1 reporter cells were incubated as described above with 0.1–80 μM FYT21 or no FYT21 as a control. Next, LDH release in 100 μl cell culture media was measured using a lactic acid dehydrogenase based *in vitro* toxicology assay kit (Sigma-Aldrich) according to manufacturer's instructions.

### Flow cytometry

THP1 reporter cells in PBS containing 0.2% BSA were incubated for 30 min on ice with conjugated mAbs directed against CD14 (BD Pharmingen, BD, USA), CD282 (Toll-like receptor-2; Hycult Biotechnology, the Netherlands) or CD284 (TLR4; Affymetrix eBioscience, Inc. USA). RAW reporter cells were incubated with conjugated mAbs directed against TLR4/MD-2 complex (BD Pharmingen). Analyses were performed on the FACSCalibur (BD) in combination with CellQuest Pro software. MFI of unstained samples were subtracted from the stained samples.

### Cell-binding assay

To investigate FYT21 binding to cell surfaces, 2 × 10^5^ THP1 reporter cells in 200 μl medium or 20 μl whole blood in 40 μl HEPES buffer were incubated with or without TAMRA-labelled FYT21 as described in the results section. After incubation, erythrocytes in whole blood were lysed using Uti-Lyse Erythrocyte Lysing Reagents (DAKO). In addition, cells were treated with trypsin (0.1–10 μg) or phosphatidylinositol-specific phospholipase C (0.1–1 U ml^−1^) for 30 min before incubation with FYT21 or the peptide was added to cells together with dermatan sulphate 36 (5–100 μg ml^−1^). The median fluorescence intensities of the various cells were measured on the FACSCalibur.

### *In vivo* models

Animal experiments were conducted according to national guidelines and approved by the local ethics committee as described above. Animals were housed under standard conditions of light and temperature, and had free access to standard laboratory chow and water.

Balb/c mice (female, 10–12 weeks; Scanbur, Denmark) were injected intraperitoneally (i.p.) with 1 mg kg^−1^ *P. aeruginosa* LPS in 100 μl PBS. PBS injection was used as a control. After 30 min, the mice were injected i.p. with 500 μg FYT21 in 100 μl 10 mM Tris. To investigate the effect of purified elastase, mice were injected subcutaneously (s.c.) with 100 μl conditioned medium of strain PAOB1 alone, or supplemented with 3 units purified *P. aeruginosa* elastase. The mice were terminated 4 h after the start of the experiment by cervical dislocation and blood was immediately collected in EDTA by cardiac puncture.

### *In vivo* imaging

Mice were injected i.p. as described above with 5 mg kg^−1^ LPS and 500 μg FYT21. At several time points, mice were anesthetised with 3.5% Isoflurane (Baxter, USA) and injected s.c. with 25 μg g^−1^ of the chemiluminescence probe L-012 (Wako Chemicals, Germany) in 50% PBS. The presence of reactive oxygen species in the mice was measured using an IVIS SPECTRUM/200 Imaging System (Caliper Life Sciences, USA). During imaging the mice were immobilized with 2% Isoflurane. Data acquisition and analyses were performed using Living Image version 4.4 (Caliper Life Sciences). Image exposure times were set automatically by the software. Results are expressed as radiance (photons per s).

### Measurement of cytokine levels

Cytokine levels in human and mouse whole-blood samples were assessed using respectively BioSource CytoSet (Invitrogen) and Cytometric Bead Array mouse inflammation kit (BD) following manufacturer's instructions.

### Electron microscopy

Detection of binding of thrombin C-terminal peptides to leukocytes in chronic venous ulcers was performed on fibrin slough fixed with 2.5% glutaraldehyde in cacodylate buffer. To investigate the binding of FYT21 to both LPS and cell membranes, THP1 cells were incubated for 30–60 min with 10 μM FYT21 and/or 100 ng ml^−1^ of LPS at 37 °C. Next, cells were harvested, centrifuged at 250*g* and resuspended in fixation solution. Finally, for detection of binding of C-terminal peptides to bacteria, *P. aeruginosa* 15159 (5 × 10^8^ ml^−1^) were incubated *ex vivo* with human plasma, acute wound fluid or chronic wound fluid for 30 min at 37 °C, washed and resuspended in fixation solution.

Sections mounted on gold grids were blocked with 50 mM glycine, incubated for 15 min with 5% goat serum in 0.2% BSA-c in PBS, pH 7.6 and then incubated overnight at 4 °C with polyclonal antibodies against VFR17 (1 μg ml^−1^), LPS (10 μg ml^−1^; LifeSpan BioScienes Inc, USA) and/or TLR4 (10 μg ml^−1^; eBioscience). Next, grids were washed, incubated for 2 h at 4 °C with 1 μg ml^−1^ of various gold labelled IgGs (BBI Solutions, UK), washed and postfixed in 2% glutaraldehyde. Finally, sections were washed in H_2_O and post-stained with 2% uranyl acetate and lead citrate. Samples were examined with a FEI/Philips CM 100 electron microscope operated at 80 kV accelerating voltage connected Olympus Veleta camera.

### Statistical analysis

To calculate the differences between *in vitro* control samples and those incubated with FYT21 or HVF18, a paired *t*-test (when comparing two groups) or a one-way ANOVA with a Dunnett's multiple comparisons test (when comparing more than two groups) was performed using Graphpad Prism version 5.03. Comparison of *in vivo* results was done using a Mann–Whitney test. A *P* value of<0.05 was considered significant.

## Additional information

**How to cite this article:** van der Plas, M. J. A. *et al. Pseudomonas aeruginosa* elastase cleaves a C-terminal peptide from human thrombin that inhibits host inflammatory responses. *Nat. Commun.* 7:11567 doi: 10.1038/ncomms11567 (2016).

## Supplementary Material

Supplementary Information Supplementary Figures 1-7 and Supplementary References

## References

[b1] Gjodsbol K. . Multiple bacterial species reside in chronic wounds: a longitudinal study. Int. Wound J. 3, 225–231 (2006). 16984578 10.1111/j.1742-481X.2006.00159.x PMC7951738

[b2] Hogsberg T., Bjarnsholt T., Thomsen J. S. & Kirketerp-Moller K. Success rate of split-thickness skin grafting of chronic venous leg ulcers depends on the presence of *Pseudomonas aeruginosa*: a retrospective study. PLoS ONE 6, e20492 (2011). 21655269 10.1371/journal.pone.0020492 PMC3105064

[b3] Vincent J. L. . Sepsis in European intensive care units: results of the SOAP study. Crit. Care Med. 34, 344–353 (2006). 16424713 10.1097/01.ccm.0000194725.48928.3a

[b4] Lyczak J. B., Cannon C. L. & Pier G. B. Lung infections associated with cystic fibrosis. Clin. Microbiol. Rev. 15, 194–222 (2002). 11932230 10.1128/CMR.15.2.194-222.2002 PMC118069

[b5] Emerson J., Rosenfeld M., McNamara S., Ramsey B. & Gibson R. L. *Pseudomonas aeruginosa* and other predictors of mortality and morbidity in young children with cystic fibrosis. Pediatr. Pulmonol. 34, 91–100 (2002). 12112774 10.1002/ppul.10127

[b6] Le Berre R. . Relative contribution of three main virulence factors in *Pseudomonas aeruginosa* pneumonia. Crit. Care Med. 39, 2113–2120 (2011). 21572326 10.1097/CCM.0b013e31821e899f

[b7] Kaminishi H. . Activation of blood clotting factors by microbial proteinases. FEMS Microbiol. Lett. 121, 327–332 (1994). 7926688 10.1111/j.1574-6968.1994.tb07121.x

[b8] Wretlind B. & Pavlovskis O. R. Pseudomonas aeruginosa elastase and its role in pseudomonas infections. Rev. Infect. Dis. 5, (Suppl 5): S998–S1004 (1983). 6419322 10.1093/clinids/5.supplement_5.s998

[b9] Schmidtchen A., Holst E., Tapper H. & Bjorck L. Elastase-producing Pseudomonas aeruginosa degrade plasma proteins and extracellular products of human skin and fibroblasts, and inhibit fibroblast growth. Microb. Pathog. 34, 47–55 (2003). 12620384 10.1016/s0882-4010(02)00197-3

[b10] Parmely M., Gale A., Clabaugh M., Horvat R. & Zhou W. W. Proteolytic inactivation of cytokines by *Pseudomonas aeruginosa*. Infect. Immun. 58, 3009–3014 (1990). 2117578 10.1128/iai.58.9.3009-3014.1990 PMC313603

[b11] Matsumoto K. Role of bacterial proteases in pseudomonal and serratial keratitis. Biol. Chem. 385, 1007–1016 (2004). 15576320 10.1515/BC.2004.131

[b12] Papareddy P. . The TFPI-2 derived peptide EDC34 improves outcome of gram-negative sepsis. PLoS Pathog. 9, e1003803 (2013). 24339780 10.1371/journal.ppat.1003803 PMC3855554

[b13] Kalle M. . Proteolytic activation transforms heparin cofactor II into a host defense molecule. J. Immunol. 190, 6303–6310 (2013). 23656734 10.4049/jimmunol.1203030 PMC3677170

[b14] Papareddy P. . Proteolysis of human thrombin generates novel host defense peptides. PLoS Pathog. 6, e1000857 (2010). 20421939 10.1371/journal.ppat.1000857 PMC2858699

[b15] Kalle M. . Host defense peptides of thrombin modulate inflammation and coagulation in endotoxin-mediated shock and Pseudomonas aeruginosa sepsis. PLoS ONE 7, e51313 (2012). 23272096 10.1371/journal.pone.0051313 PMC3521733

[b16] Choi K. Y., Chow L. N. & Mookherjee N. Cationic host defence peptides: multifaceted role in immune modulation and inflammation. J. Innate Immun. 4, 361–370 (2012). 22739631 10.1159/000336630 PMC6741489

[b17] Hilchie A. L., Wuerth K. & Hancock R. E. Immune modulation by multifaceted cationic host defense (antimicrobial) peptides. Nat. Chem. Biol. 9, 761–768 (2013). 24231617 10.1038/nchembio.1393

[b18] Pasupuleti M., Schmidtchen A. & Malmsten M. Antimicrobial peptides: key components of the innate immune system. Crit. Rev. Biotechnol. 32, 143–171 (2011). 22074402 10.3109/07388551.2011.594423

[b19] Hancock R. E. & Sahl H. G. Antimicrobial and host-defense peptides as new anti-infective therapeutic strategies. Nat. Biotechnol. 24, 1551–1557 (2006). 17160061 10.1038/nbt1267

[b20] Bowdish D. M. . Impact of LL-37 on anti-infective immunity. J. Leukoc. Biol. 77, 451–459 (2005). 15569695 10.1189/jlb.0704380

[b21] Sato S. . Synergy and cross-tolerance between toll-like receptor (TLR) 2- and TLR4-mediated signaling pathways. J. Immunol. 165, 7096–7101 (2000). 11120839 10.4049/jimmunol.165.12.7096

[b22] Penc S. F. . Dermatan sulfate released after injury is a potent promoter of fibroblast growth factor-2 function. J. Biol. Chem. 273, 28116–28121 (1998). 9774430 10.1074/jbc.273.43.28116

[b23] Baranska-Rybak W., Sonesson A., Nowicki R. & Schmidtchen A. Glycosaminoglycans inhibit the antibacterial activity of LL-37 in biological fluids. J. Antimicrob. Chemother. 57, 260–265 (2006). 16387752 10.1093/jac/dki460

[b24] Kielland A. . In vivo imaging of reactive oxygen and nitrogen species in inflammation using the luminescent probe L-012. Free Radic. Biol. Med. 47, 760–766 (2009). 19539751 10.1016/j.freeradbiomed.2009.06.013

[b25] Butenas S. & Mann K. G. Blood coagulation. Biochemistry (Mosc) 67, 3–12 (2002). 11841335 10.1023/a:1013985911759

[b26] Bardoel B. W. . Pseudomonas evades immune recognition of flagellin in both mammals and plants. PLoS Pathog. 7, e1002206 (2011). 21901099 10.1371/journal.ppat.1002206 PMC3161968

[b27] Madani F., Lindberg S., Langel U., Futaki S. & Graslund A. Mechanisms of cellular uptake of cell-penetrating peptides. J. Biophys. 2011, 414729 (2011). 21687343 10.1155/2011/414729 PMC3103903

[b28] Yeaman M. R. & Yount N. Y. Mechanisms of antimicrobial peptide action and resistance. Pharmacol. Rev. 55, 27–55 (2003). 12615953 10.1124/pr.55.1.2

[b29] Kasetty G. . Structure-activity studies and therapeutic potential of host defense peptides of human thrombin. Antimicrob. Agents Chemother. 55, 2880–2890 (2011). 21402837 10.1128/AAC.01515-10 PMC3101415

[b30] Marples M. The Ecology of the Human Skin Thomas (1965).

[b31] Grice E. A. . A diversity profile of the human skin microbiota. Genome Res. 18, 1043–1050 (2008). 18502944 10.1101/gr.075549.107 PMC2493393

[b32] Grice E. A. & Segre J. A. The skin microbiome. Nat. Rev. Microbiol. 9, 244–253 (2011). 21407241 10.1038/nrmicro2537 PMC3535073

[b33] Kasetty G. . The C-terminal sequence of several human serine proteases encodes host defense functions. J. Innate Immun. 3, 471–482 (2011). 21576923 10.1159/000327016

[b34] Snitkin E. S. & Segre J. A. Pseudomonas aeruginosa adaptation to human hosts. Nat. Genet. 47, 2–3 (2015). 25547595 10.1038/ng.3172

[b35] Adekoya O. A. & Sylte I. The thermolysin family (M4) of enzymes: therapeutic and biotechnological potential. Chem. Biol. Drug. Des. 73, 7–16 (2009). 19152630 10.1111/j.1747-0285.2008.00757.x

[b36] Schmidtchen A., Frick I. M., Andersson E., Tapper H. & Bjorck L. Proteinases of common pathogenic bacteria degrade and inactivate the antibacterial peptide LL-37. Mol. Microbiol. 46, 157–168 (2002). 12366839 10.1046/j.1365-2958.2002.03146.x

[b37] Schmidtchen A. Degradation of antiproteinases, complement and fibronectin in chronic leg ulcers. Acta Derm. Venereol. 80, 179–184 (2000). 10954207 10.1080/000155500750042925

[b38] Toder D. S., Gambello M. J. & Iglewski B. H. Pseudomonas aeruginosa LasA: a second elastase under the transcriptional control of lasR. Mol. Microbiol. 5, 2003–2010 (1991). 1766376 10.1111/j.1365-2958.1991.tb00822.x

[b39] Lehrer R. I., Rosenman M., Harwig S. S., Jackson R. & Eisenhauer P. Ultrasensitive assays for endogenous antimicrobial polypeptides. J. Immunol. Methods 137, 167–173 (1991). 1901580 10.1016/0022-1759(91)90021-7

[b40] Ulevitch R. J. The preparation and characterization of a radioiodinated bacterial lipopolysaccharide. Immunochemistry 15, 157–164 (1978). 417017 10.1016/0161-5890(78)90144-x

[b41] Schmidtchen A., Frick I. M. & Bjorck L. Dermatan sulphate is released by proteinases of common pathogenic bacteria and inactivates antibacterial alpha-defensin. Mol. Microbiol. 39, 708–713 (2001). 11169110 10.1046/j.1365-2958.2001.02251.x

