# Supplementary material for: Pseudomonas aeruginosa elastase cleaves a C-terminal peptide from human thrombin that inhibits host inflammatory responses
Source: Nat Commun. 2016 May 16;7:11567. doi: 10.1038/ncomms11567 (PMC4873665; doi:10.1038/ncomms11567)
Supplement: Supplementary Information — Supplementary Figures 1-7 and Supplementary References [file ncomms11567-s1.pdf]

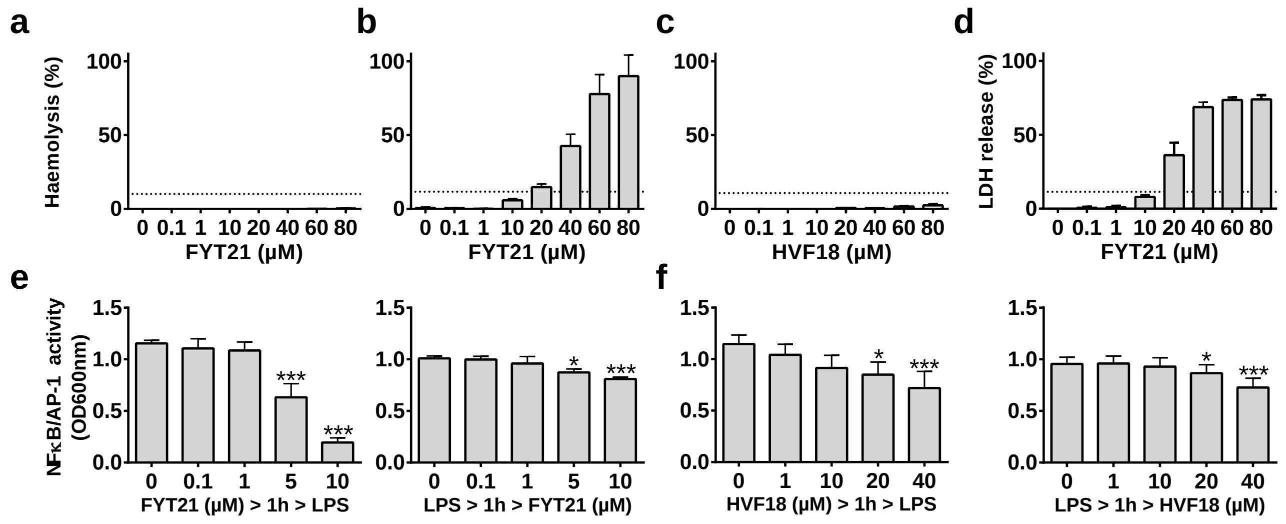

Supplementary Figure 1 Haemolysis, LDH-release and cell activation by FYT21 and HVF18. Haemolysis of erythrocytes present in (**a**) 25% or (**b, c**) 0.5% whole blood by FYT21 or HVF18. (**d**) FYT21-induced LDH-release in THP1 cell cultures. Results are means ± s.e.m. of 3-4 experiments. Cells were preincubated with (**e**) FYT21 or LPS for 1 h followed by stimulation with respectively LPS or FYT21 overnight or (**f**) HFV18 or LPS for 1 h followed by addition of respectively LPS or HVF18 overnight and NFκB/AP-1 activity was measured. Results are means ± s.e.m. of 4-7 experiments. Values are significantly (*p<0.05 and ***p<0.0005) different from the controls as analysed using a one-way ANOVA with Dunnett's multiple comparisons test.

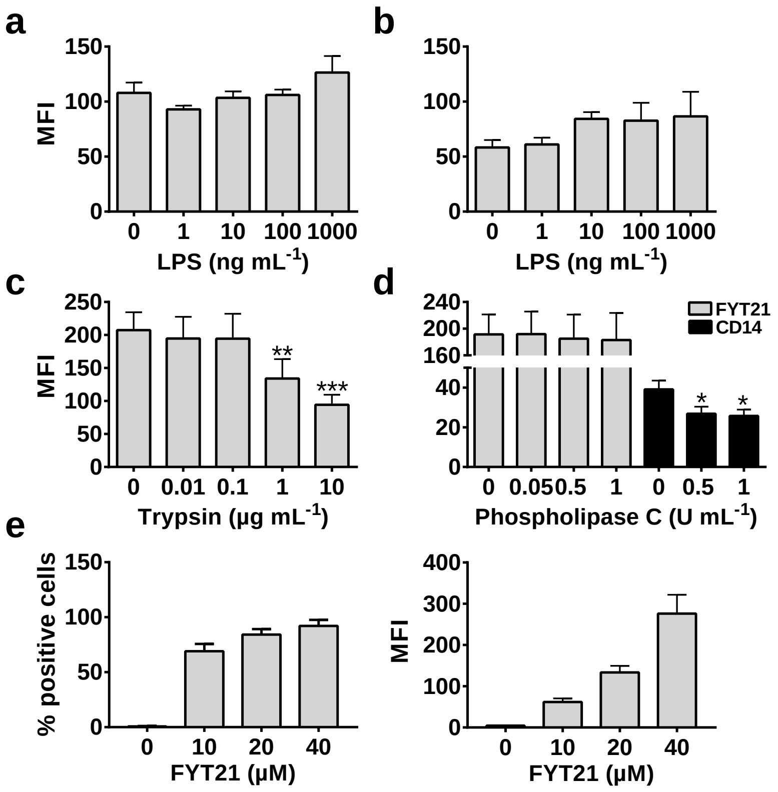

Supplementary Figure 2 Binding of FYT21 to cells under various conditions. Binding of 10 µM TAMRA-labelled FYT21 to THP1 cells in the presence of a range of LPS without (**a**) or with (**b**) 10% heat-inactivated fetal bovine serum in the medium. Effect of preincubation of THP1 cells with a range of trypsin (**c**) or phosphatidylinositol-specific phospholipase C (**d**) for 30 min at respectively 37 °C and 4 °C on the binding of 10 µM FYT21. CD14 expression was used as a positive control for phospholipase C activity. Results are means ± s.e.m. of 4-6 experiments. Values are significantly (*p<0.05, **p<0.005 and ***p<0.0005) different from the controls as analysed using a one-way ANOVA with Dunnett's multiple comparisons test. (**e**) Binding of TAMRA-labelled FYT21 to human platelets. Results, expressed as the percentage FYT21 positive cells and median fluorescence intensity (MFI), are means ± s.e.m. of 4 experiments.

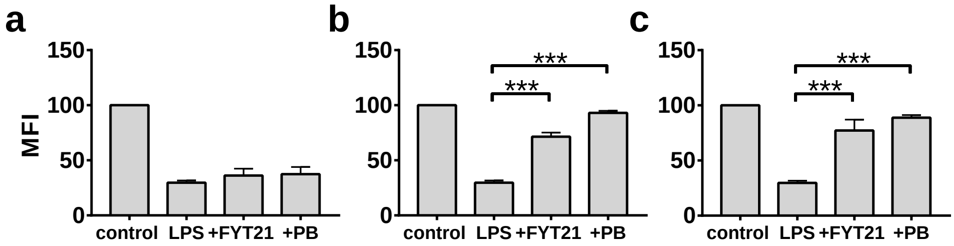

Supplementary Figure 3 Effect of preincubation on the effect of FYT21 on LPS-induced dimerization. RAW cells were incubated for 30 min with (**a**) LPS (1 µg mL^{-1}) or (**b**) FYT21 (10 µM) followed by a second 30 min incubation after the addition of respectively FYT21 and LPS, or (**c**) LPS and FYT21 were preincubated for 30 min before addition to the cells. Polymyxin B (PB; 50 µg mL^{-1}) was used as a control. The effect of FYT21 and PB on LPS-induced TLR4 dimerization is expressed as a percentage relative to the control (which was set to 100%). Results are means ± s.e.m. of 4 experiments. Values are significantly (***$p<0.0005$) different from the controls as analysed using a one-way ANOVA with Dunnett's multiple comparisons test.

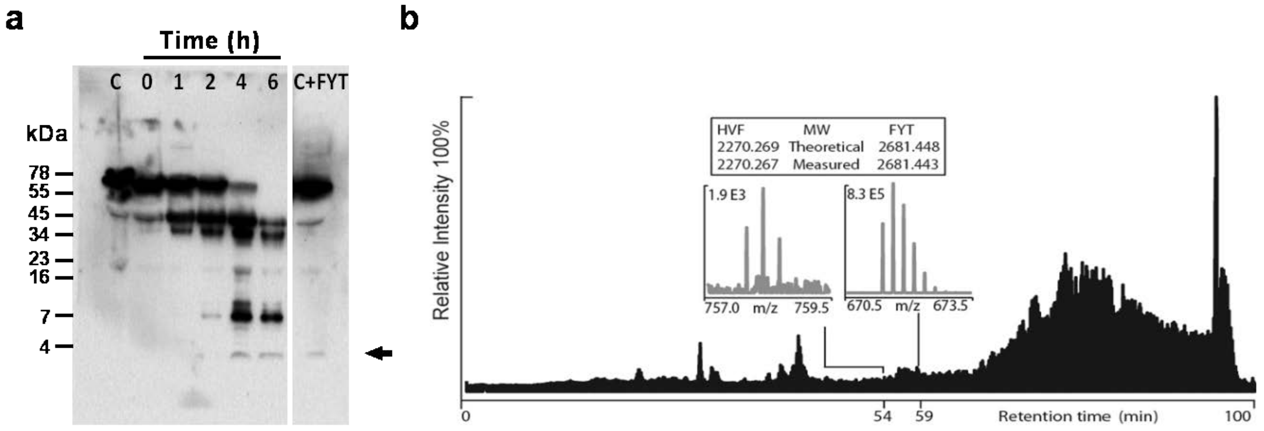

Supplementary Figure 4 FYT21 is formed in whole blood in the presence of *P. aeruginosa* elastase. (**a**) Whole blood was incubated with 10% conditioned medium for several time points at 37 °C, and plasma was collected and analysed using western blot. The arrow indicates peptides of similar molecular mass as FYT21 (right lane). (**b**) LC-MS/MS chromatogram of affinity purified 4 h sample (in black). The reversed phase gradient separated HVF18 and FYT21 at respectively 54 and 59 min. The isotopic distribution of triple-charged HVF18 (m/z 757.7625) and quadruple-charged FYT21 (m/z 671.3670) is shown in the inserts.

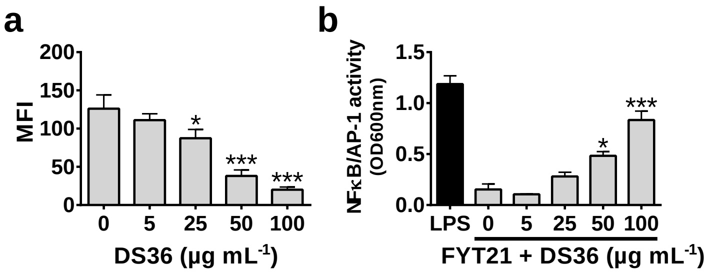

Supplementary Figure 5 Effect of dermatan sulphate 36 on the activity of FYT21. (**a**) Binding of 10 µM FYT21 to THP1 cells after 30 min incubation with dermatan sulphate (DS36) was analysed by FACS. (**b**) LPS-induced NFκB/AP-1 activity in the presence of 10 µM FYT21 and various concentrations of DS36. Results are means ± s.e.m. of 3 experiments. Values are significantly (*$p<0.05$ and ***$p<0.0005$) different from the controls as analysed using a one-way ANOVA with Dunnett's multiple comparisons test.

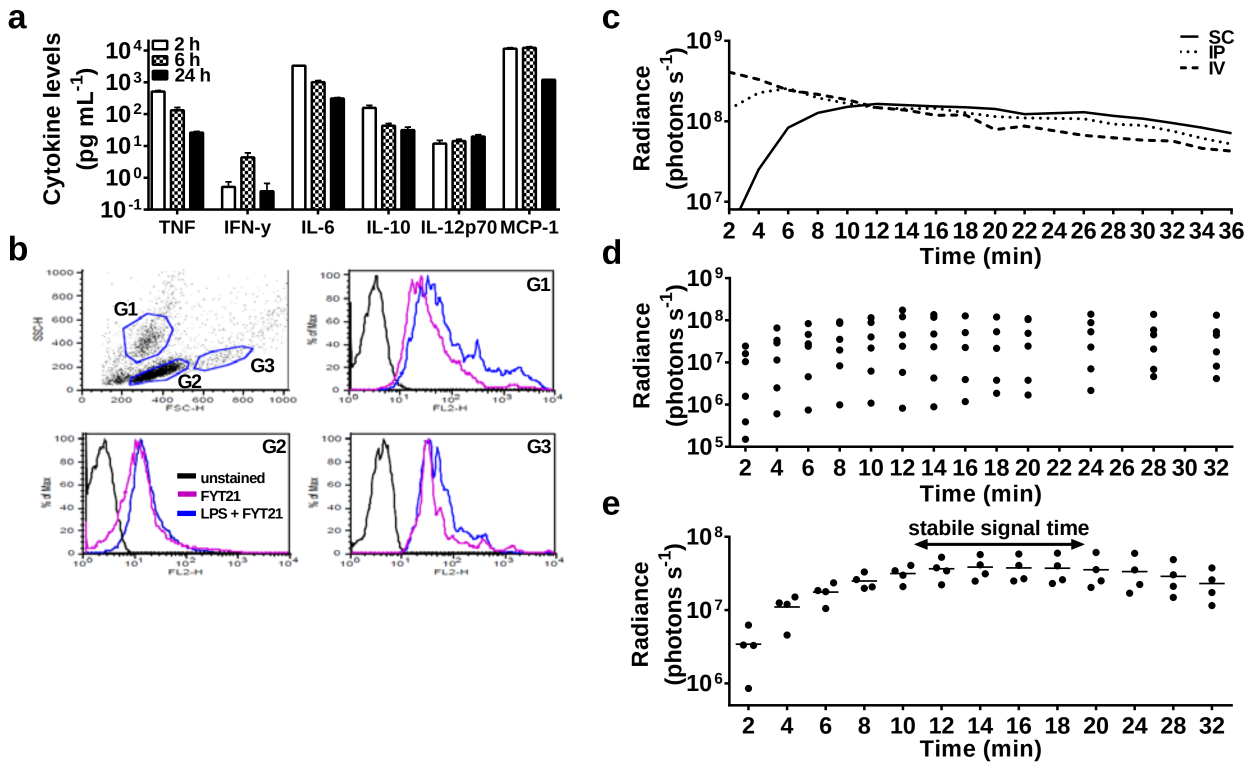

Supplementary Figure 6 *In vivo* analysis of LPS and FYT21. (**a**) Mice were injected intraperitoneally (i.p.) with 1 mg kg^{-1} LPS and blood was collected in EDTA after 2, 6 or 24 h by cardiac puncture after cervical dislocation. (**b**) Representative examples of a FACS plot and histograms showing binding of FYT21 to intraperitoneal cells (gates G1-3 displaying neutrophils, lymphocytes and mononuclear phagocytes), 2 h after i.p. injection of LPS or PBS which was followed 30 min later with TAMRA-labelled FYT21. (**c**) Representative examples of ROS production 4 h after injection of mice with LPS, measured using an IVIS SPECTRUM/ 200 imaging system after subcutaneous (s.c.), i.p. or intravenous (i.v.) injection with the reactive oxygen species probe L-012. Mice were injected i.p. with (**d**) 1 mg kg^{-1} or (**e**) 5 mg kg^{-1} LPS followed after 4 h with anesthetising and s.c. injection of L-012. The radiance was measured every two min for up to 32 min. The results are of representative experiments using 5 and 4 mice respectively.

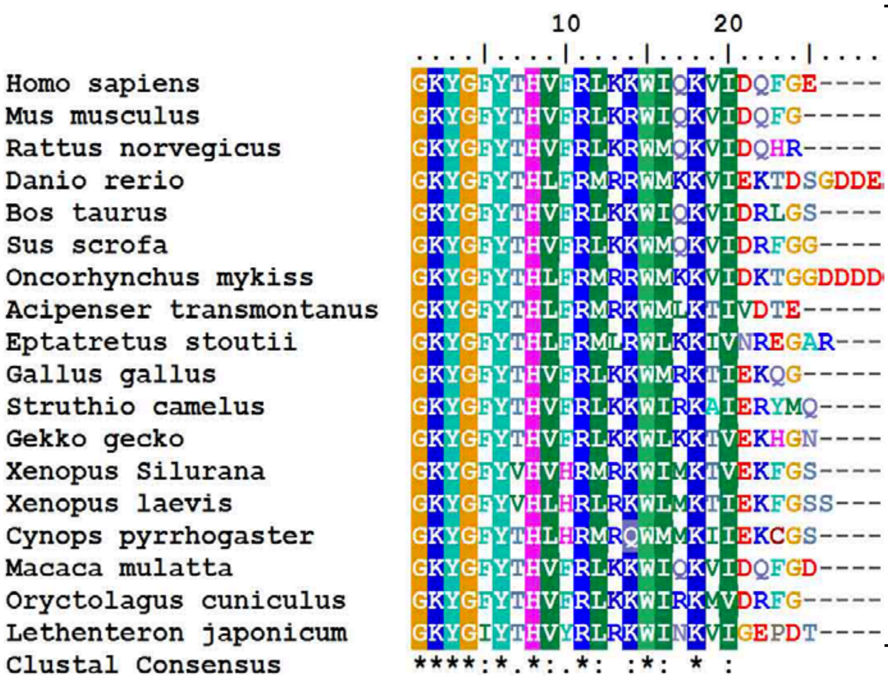

Charge	Agadir	µHrel
+3	2.85	0.43
+4	3.56	0.4
+5	2.69	0.28
+2	3.67	0.31
+5	3.49	0.42
+5	2.52	0.36
+2	3.11	0.3
+2	2.97	0.28
+6	18.20	0.33
+6	1.95	0.28
+6	4.80	0.4
+6	2.65	0.3
+5	0.69	0.34
+5	2.65	0.42
+4	2.93	0.43
+3	2.85	0.43
+6	3.81	0.39
+3	2.00	0.34

Supplementary Figure 7 The C-terminal part of thrombin is highly conserved. Alignment of the C-terminal part of thrombin of various species and corresponding biophysical parameters are shown. A positive charge, helical propensity (Agadir)[1] and a relative hydrophobicity (µHrel) of 0.3-0.5 characterises many host defence peptides (see [2,3] for further information).

Supplementary references

1. Lacroix, E., Viguera, A. R., and Serrano, L. Elucidating the folding problem of alpha-helices: local motifs, long-range electrostatics, ionic-strength dependence and prediction of NMR parameters. *J. Mol. Biol.* **284**: 173–191 (1998).
2. Pasupuleti, M., Walse, B., Svensson, B., Malmsten, M., & Schmidtchen, A. Rational design of antimicrobial C3a analogues with enhanced effects against Staphylococci using an integrated structure and function-based approach. *Biochem*. **47**: 9057-9070 (2008).
3. Kasetty, G. *et al*. The C-terminal sequence of several human serine proteases encodes host defense functions. *J. Innate Immun*. **3**: 471-482 (2011).
